# Factors related to fertility desire among female sex workers living with HIV in the Dominican Republic

**DOI:** 10.1186/s12905-018-0613-1

**Published:** 2018-07-03

**Authors:** Dana Cernigliaro, Clare Barrington, Martha Perez, Yeycy Donastorg, Deanna Kerrigan

**Affiliations:** 1 The Johns Hopkins Bloomberg School of Public Health, 624 N Broadway HH 257, Baltimore, MD 21205 USA; 2 The University of North Carolina Gillings School of Global Public Health, Chapel Hill, NC USA; 3 Instituto Dermatologico y Cirugia de la Piel, Santo Domingo, Dominican Republic

**Keywords:** Dominican Republic, HIV/AIDS, Female sex work, Fertility desire, Stigma

## Abstract

**Background:**

Female sex workers living with HIV are at increased risk for negative health outcomes and multiple levels of stigma. However, there is limited research on female sex workers living with HIV and even less focused on reproductive health.

**Methods:**

We analyzed data using logistic regression from a cohort of 247 female sex workers of reproductive age living with HIV in Santo Domingo, Dominican Republic to assess factors associated with fertility desire.

**Results:**

Most participants had children (93.1%; mean: 2.8; range: 1,8) and 28.3% reported fertility desire. Bivariate regression analysis uncovered that participants who desired children were less likely to report being on antiretroviral treatment and more likely to have a detectable viral load. Multivariate regression results showed participants who desired more children were: less likely to be older, have higher levels of HIV-related internalized stigma, have a history of pregnancy loss, have fewer children and have a perception that their partner has negative feelings about pregnancy.

**Conclusions:**

Individual and interpersonal characteristics were found to be associated with fertility desire in this study. Additional in-depth research is needed to understand how the role of stigma, partner dynamics and reproductive history as it relates to fertility desire, in order to ensure the reproductive health and wellbeing of this population.

## Background

The global prevalence of HIV among female sex workers is estimated at 11.8% and is estimated at 13.5 times that of the general female population (women aged 15–49) in low and middle-income countries [1]. Despite this significant burden of disease there is limited research specifically on female sex workers living with HIV, particularly with regard to sexual and reproductive health.

Among marginalized women, including women living with HIV, motherhood has been described as a way to feel valued by the woman herself and within her social or familial context [2, 3]. With the discovery and accessibility of antiretroviral treatment (ART), both length and quality of life, as well as prevention of mother-to-child transmission (MTCT) of HIV was possible [4], impacting family planning decisions. Many women living with HIV globally desire children and in some regions this desire is similar to women in the general population [5]. Fertility desire among women living with HIV has been associated with younger age, fewer current children, increased desire for motherhood, having lost a child, how healthy they feel and stigma [5]. More consistently, however, culture, social expectation and the importance of a woman’s identity as a mother are found as strongly influential across settings [2, 6, 7]. Women have increased fertility desire in cultures that place high importance, expectation and value on fertility or where women without children face stigma and discrimination [5, 8]. Internalized and societal stigma have also been found to influence fertility desire [2, 7]. Women with a higher degree of HIV-related internalized stigma were more likely to want children, which would conceal their positive status and improve perceived self-worth, while those with higher HIV-related social stigma were less likely to want children to avoid societal judgment and criticism from others [5]. Having children was described as providing a sense of fulfillment, increased self-esteem, and a reason to keep living [2, 3, 6]. However, women also describe concern about the inability to care for children due to an HIV-related sickness or for fear of mother-to-child transmission of HIV [8]. Therefore, many times women find themselves making childbearing decisions amidst tension between self-image, culture, social expectation and fears about health for themselves and future children [3].

Children and pregnancy have been found to play an important role among female sex workers as well, where sex workers may seek pregnancy through sex work [1, 9, 10], exit sex work due to pregnancy [9, 10], or enter into sex work to support children [10, 11]. Fertility intentions among female sex workers have been linked to both demographic and socio-environmental factors related to their relationships and places of work [12, 13]. Sex workers are at increased risk for unintended pregnancy, abortion [11], may continue sex work throughout pregnancy, [14] and face barriers to health services [15], increasing risk for poor maternal-child health outcomes.

The emerging literature on female sex workers living with HIV has documented significant health concerns including increased risk for other sexually transmitted infections, violence, poor mental health outcomes, HIV care interruption and multiple forms of stigma and discrimination [8, 11, 16]. Despite the call for non-discriminatory services by UNAIDS [17], female sex workers living with HIV have encountered barriers to care due to both occupational and HIV-related stigma. They were more likely to have reported experiencing humiliation, being demeaned by health workers [18] having felt socially isolated, being refused medical care or feared seeking health services compared to female sex workers without HIV [19].

In the Dominican Republic (DR), the exchange of sex for money among those over 18 years old is not explicitly criminalized and organizations exist to educate and empower female sex workers. HIV prevalence among female sex workers is estimated at 4 to 5% [20, 23] but varies depending on region, reach and intensity of prevention intervention coverage. Family and childbearing are culturally important, forming the basis of social support, particularly in low-income populations [21]. The DR has strict abortion laws, prohibiting all abortions (The Penal Code of 1948, section 317) except in situations where the woman’s life it at risk. Those who perform, consent to or cause their own abortion face harsh penalties, particularly medical professionals [22]. Reproductive health services for the general population in the DR continues to be an issue. The maternal mortality ratio in the DR (159/100,000 live births) is higher than other countries in Latin America and the Caribbean and most affects underserved, low-income younger women [23]. Modern contraceptive prevalence is high for the general population of the DR at 73% with sterilization accounting for almost half of all the methods used [23]. For women living with HIV/AIDS (WLHA), access to non-discriminatory services is especially important in order to access to education and services for themselves as well as for their partners and their children. ARTs for adults were available in 60% of hospitals, though they were not always fully stocked. In terms of contraception services, only 36% of providers in Integral Care Units interviewed in the DR said they provided contraceptive counseling to WLHA and of those that responded, only 41% offered contraceptive services [24]. A study of health providers in the DR who counseled WLHA found that providers most commonly recommended consistent condom use for contraception. However, a large proportion of providers who counseled WLHA on family planning believed that WLHA should not have children and about 36% said they emphasized sterilization. Qualitative results from this study uncovered that discrimination against WLHA by providers was observed in subtle and more aggressive ways [24].

Knowing that both women living with HIV and female sex workers have and desire children while facing increased health risks and significant barriers to care, it is essential that we understand more about pregnancy and childbearing among this population. This study aim is to understand factors associated with fertility desire among female sex workers living with HIV in Santo Domingo, DR.

## Methods

This study used a cross-sectional analysis of baseline data from a longitudinal intervention study, named *Abriendo Puertas (*Opening Doors*). Abriendo Puertas* was guided by formative research, [25] and aimed to assess a multi-level intervention to promote HIV protective behaviors and foster adherence to care and treatment among female sex workers living with HIV in Santo Domingo. The intervention included individual counseling and education, peer navigation, clinical provider sensitivity training and community mobilization and aimed to promote HIV care and preventive behaviors on HIV outcomes and behaviors [16].

### Study sample & recruitment

Female sex workers were defined as women who reported having exchanged sex for money in the last month. Participants were at least 18 years old, spoke Spanish, and reported HIV infection, confirmed prior to enrollment by an HIV test (Retrocheck). Recruitment occurred predominantly through peer navigators both in the community and in HIV clinics, with a small minority of participants recruited via participant referrals. Peer navigators were current/former sex workers with experience with HIV outreach, prevention and support. Enrollment occurred from November 2012 to February 2013, resulting in a sample size of 268 participants. This paper focuses on participants of reproductive age (15 to 46 years old), totaling 247 participants. Due to the high number of participants who reported permanent contraception and fertility desire, analysis was run on the full sample and on those who reported not having had a permanent contraceptive procedure (*n* = 125). If participants indicated that they had had a tubal ligation or a hysterectomy and reported the year of the procedure were determined as having had a permanent contraceptive method. Studies in the DR have found that women living with HIV sometimes did not understand the permanence of sterilization [26] or reported regret, which is of concern [27].

### Survey description

The baseline socio-behavioral survey included several sections including demographics, employment, HIV testing experience, disclosure, health care services, clinic and provider dynamics, ART experiences, sexual behavior, social support, reproductive and sexual health, HIV intervention exposure, HIV knowledge, alcohol/drug use, violence, community engagement and stigma/discrimination.

### Data collection

Surveys were conducted in Spanish within private offices of the HIV Vaccine Research Unit by female Dominican field staff. All surveys were de-identified and kept in a locked cabinet at Instituto Dermatologico y Cirugia de Piel Dr. Humberto Bogart Diaz (IDCP). The survey was entered into an electronic database by trained staff onto a password secured computer and external hard drive. Viral load was assessed through blood samples at the Dominican National Reference Laboratory using polymerase chain reaction (PCR) methods.

### Ethics and collaborative partners

This study partnered with IDCP, and the non-governmental organizations Movimiento de Mujeres Unidas (MODEMU), a sex worker rights group, and Centro de Orientacion e Investigacion Integral (COIN), an HIV prevention organization. Participants provided oral consent rather than written consent to protect confidentiality of a highly stigmatized population group. All participants were offered 10 USD for completion. The Johns Hopkins Bloomberg School of Public Health, University of North Carolina and IDCP Institutional Review Boards approved the study.

## Measures

### Dependent variable

The primary outcome was assessed using the question, “Would you like to have (more) children in the future?”. Response options were “yes”, “no” or “maybe”. Only 2 respondents responded “maybe” and were grouped with “yes” responses since these participants were open to having children.

### Independent variables

Along with background literature, the Theory of Planned Behavior, an established behavioral theory that explores health behavior, beliefs, attitudes and intentions, guided variable selection [28] and has been used in studies related to fertility intention [5]. Key independent variables included sociodemographics (age, education and number of children), viral load, positive perception of HIV and pregnancy was determined if the participant agreed with the statement “If an HIV-positive woman wants to get pregnant, it is good to try to get pregnant”. Participants were asked how many times they have become pregnant since diagnosed with HIV, clarifying that “pregnant” included any pregnancy that resulting in a miscarriage, termination of pregnancy, stillbirth, or live birth. They were also asked how many times, since HIV diagnosis they have given birth to a live baby. Pregnancy loss was measured by pregnancies not resulting in a live birth, which may also include report of illegal abortions. Other key variables include perceived provider support for pregnancy, perceived partner feelings about pregnancy and internalized stigma scales (HIV and sex work related). Perceived partner feelings about pregnancy was chosen for the final model over civil status due to prior research which found a strong influence of partner expectations and support on pregnancy intent [5]. Child loss was defined by the difference of reporting the number of live births they have had since HIV diagnosis and number of current children they have, although how the loss occurred was not reported. Two separate internalized stigma scales for sex work and HIV used adapted measures from those developed by Berger et al. [29] and Zelaya et al. [30] guided by Earnshaw’s HIV Stigma Framework [31]. Statements such as “Having HIV makes you feel like a bad person” or “You feel ashamed that you have HIV” among others were included in the HIV stigma scale, while the sex work stigma scale asked similar questions about sex work. Response options included: 1 = totally disagree, 2 = disagree, 3 = agree, 4 = totally agree, 88 = don’t know and 99 = refuse to answer. Answers of “don’t know” and “refuse to answer” were coded as 2.5 to retain those responses but neutralize their weight, though analysis was run with and without the neutral coding to ensure no significant differences. Data reduction occurred through principal components analysis. Eigenvalues of ≥1 were considered, along with scree plots and parallel factor analysis results, followed by a test of normality. Factors were rotated and if factor loadings were less than 0.4 and uniqueness was greater than 0.5, the factors were dropped. Cronbach’s alpha measured the internal consistency of the final scales, which were 0.88 for the HIV stigma scale (retaining 7/8 items) and 0.91 for sex work stigma scale (retaining 12/15 items). Final items were averaged across participant to create the final composite scores. Further detail on the study design and measures are found in the *Abriendo Puertas* baseline paper [16].

### Data analysis

Exploratory data analysis was conducted and categories were created according to data distribution. Original surveys were re-checked to identify and account for missing data and ensure data integrity, minimizing missing values. T-tests and chi-square tests, as appropriate, were calculated between independent variables and the outcome. Bivariate logistic regression was conducted for each independent variable. The model for multivariate logistic regression was built in response to unadjusted relationships (inclusion was determined at < 0.10 *p*-value), background literature and theoretical relevance. Multicollinearity was assessed for final models. If two variables were correlated, variables with greater theoretical relevance or associations found in the literature were chosen. The final model was built using multiple iterations through a traditional stepwise approach, controlling for age and education. The Akaike Information Criterion [32] was calculated for each nested model and log likelihood tests were assessed. The final model was chosen with the most parsimonious fit (lowest Akaike and log likelihood with the greatest theoretical relevance). The fit of the final model was determined by a Hosmer-Lemeshow goodness-of-fit test [33].

## Results

Descriptive characteristics are highlighted in Table 1. The mean age of participants was 34 years (range: 18,49 years) with most reporting a current primary male spouse, live-in or regular partner (201/247, 81.4%). Almost all participants had some education (243/247, 98.4%) with most having only a primary education (153/247, 61.9%).

**Table 1:** Descriptive characteristics of the study population (*n* = 247)

Socio-demographic characteristics	N	%	Mean	Range
Age			34.3	18,46
Civil status				
Single/Wid/Div	46/247	18.6		
Current partner	201/247	81.4		
Education (ever)	243/247	98.4		
Primary	153/247	61.9		
Secondary/tertiary	94/247	38.1		
Residence				
Santo Domingo	192/247	77.7		
Other	55/247	22.3		
Behavioral characteristics				
Sex work entry age (years)			20	10,46
Average income/salida (pesos)			889^a^	200,4000
Work Locations				
Establishment	149/247	59.6		
Street	140/247	55.3		
Partner conflict	89/247	36.0		
HIV and sexual health				
Years since HIV diagnosis (*n* = 245)			5.8	0,18
Current ART	177/247	71.7		
Detectable viral load (*n* = 243)	131/243	53.9		
Any pregnancy prevention	200/247	81.0		
Contraception methods				
Oral contraceptive	11/247	4.5		
Injectables (Depo-Provera or Nuristerate)	6/247	2.4		
IUD	3/247	1.2		
Diaphragm	3/247	1.2		
Reported permanent contraception (hysterectomy or tubal ligation)	121/247	49.2		
Consistent condom use	157/247	64.1		
Stigma scales				
HIV-internalized stigma			2.42	1,4
Sex work-internalized stigma			2.38	1,4

^a^Approximately 20 USD

The mean age for sex work entry was 20 years (range: 10,46 years). Most of participants engaged in street-based (140/247, 55.3%) and/or establishment-based (eg. club, bar, hotel, *colmadon* or *billar*) (149/247, 59.6%) sex work. Participants could respond to more than one work location. The average income from each *salida* (sex work date) was 890 Dominican pesos (approximately 20 USD), (range: 200,4000 Dominican pesos/*salida*). About a third (89/247, 36.0%) reported a conflict, or disagreement, with a partner (last 6 months). About a quarter of those (23/89, 25.8%) reported that conflicts were physically, mentally, verbally, emotionally and/or economically abusive- where their partner controls access to resources, creating economic dependency and coercion.

On average participants self-reported living with HIV for 6 years (range: < 1 year,18 years). While most reported current antiretroviral therapy (ART) use (177/247, 71.7%), about 46.0% had an undetectable viral load (< 50 copies/mL) and 74/193 (38.3%) reported ever stopping ART. Many participants reported contraceptive use specifically for pregnancy prevention (past 6 months) (200/247, 80.9%), and consistent condom use with all partners was 64.1% (157/247). However, current non-permanent self-reported contraceptive methods were low, including oral contraceptives at 4.5% (11/247), Injectables at 2.4%, and IUD and Diaphragm independently at 1.2% (3/247). About half (121/247, 49.2%) reported a permanent contraceptive procedure (sterilization: 115/247, 46.6%, hysterectomy: 8/247, 3.2%). One participant reported both tubal ligation and a hysterectomy but was only counted once in the permanent contraceptive measure. Stigma scale averages revealed HIV-related internalized stigma at 2.42/4.0 and sex work internalized stigma at 2.38/4.0.

Table 2 describes fertility and childbearing characteristics. Almost all participants had been pregnant (236/247, 95.5%, mean: 4.4, range: 1,12) and 93.1% (230/247) reported at least one child (mean: 2.8, range: 1,8). About 64.0% (152/236) of participants reported at least one pregnancy loss. Over a third of participants reported a pregnancy since HIV diagnosis (91/247, 36.8%, mean: 1.6, range: 1,5).

**Table 2:** Fertility and childbearing characteristics (*n* = 247)

	Number	%	Mean	Range
Currently have children	230/247	93.1		
Number of children (*n* = 230)			2.8	1,8
Child loss (ever)	51/247	20.7		
Ever pregnant	236/247	95.5		
Number of pregnancies (*n* = 236)			4.4	1,12
Any pregnancy loss	152/236	64.4		
Pregnant since HIV diagnosis	91/247	36.8		
Number of pregnancies (*n* = 91)			1.6	1,5
Any pregnancy loss	32/91	35.2		
Fertility desire	70/247	28.3		
Number of children desired (*n* = 68)			1.6	1,5
Reported permanent contraception	24/70	34.3		
Currently pregnant	5/247	2.0		
Negative perception of pregnancy and HIV	136/247	55.1		
^a^Partner would be upset about pregnancy	30/201	14.9		

^a^Among those reporting a partner

Of those pregnant after HIV diagnosis, about a third (32/91, 35.2%) reported a pregnancy loss. Five women reported currently being pregnant (5/247, 2.0%) and three were unsure (3/247, 1.2%). Almost 30% of the participants (70/247) desired more children (range: 1,5; average: 1.6). Among those wanting children, 34.3% (24/70) also reported a permanent contraceptive procedure. While many indicated fertility desire, 55.0% (136/247) had a negative perception of HIV and pregnancy. Among those reporting a partner, 14.9% (30/201) felt a pregnancy would upset their partner.

Table 3 highlights bivariate logistic regression results for the total sample and for those who reported not having a permanent contraception procedure. In the bivariate analysis, older age (OR: 0.88; 95% CI: 0.8, 0.92), having more children (OR: 0.5; 95% CI: 0.39,0.64), living with HIV longer (OR: 0.89; 95% CI: 0.83, 0.96) and current ART use (OR: 0.46; 95% CI: 0.25,0.82) were all negatively associated with fertility desire in the total sample. Having a detectable viral load (OR: 2.16; 95% CI: 1.21,3.87) was positively associated with fertility desire. Civil status, education, alcohol/drug use, knowledge of mother-to-child transmission and years in sex work were not significantly associated with fertility desire. Participants reporting a positive perception of pregnancy and HIV were 6.14 times more likely to desire children compared to those who did not (OR: 6.14, 95% CI: 3.19,11.79) and those who reported a pregnancy loss were less likely to want children than those that haven’t (OR: 0.40, 95% CI: 0.23,0.71). There was marginally non-significant association between child loss and fertility desire (OR: 0.67; 95% CI: 0.40,1.1). Participants who perceived a pregnancy would upset their partners had lower odds of fertility desire compared to perceived support (OR: 0.10; 95% CI: 0.02,0.45). Lastly, participants reporting higher HIV-related internalized stigma were 1.6 times more likely per unit increase in the scale to want more children (OR: 1.60, 95% CI: 1.26,5.7).

**Table 3:** Bivariate associations with fertility desire among female sex workers living with HIV

Sociodemographics	Total study sample (*n* = 247)		Participants not reporting permanent contraception (*n* = 125)	
	OR	95% CI	OR	95% CI
Age	0.88***	(0.84,0.92)	0.90***	(0.86,0.95)
Civil status (partner)	1.00	–	1.00	–
Single/wid/div	1.00	(0.49,2.05)	0.79	(0.57,1.08)
Education	1.12	(0.63,1.97)	0.92	(0.44,1.91)
Number of children	0.50***	(0.39,0.64)	0.51***	(0.36,0.72)
HIV and sex work				
Years HIV positive (n = 245)	0.89*	(0.83 0.96)	0.91	(0.82,1.01)
Age first engaged in sex work	0.96	(0.93,1.01)	0.91	(0.82,1.01)
Partner conflict	1.61	(0.92,2.85)	1.76	(0.83,3.75)
Current ART	0.46*	(0.25,0.82)	0.51	(0.23,1.09)
Viral load (ref = undetectable)	2.16*	(1.21,3.87)	2.30*	(1.07,4.92)
Sexual and reproductive health				
Perception of pregnancy and HIV (ref = negative)	6.14***	(3.19,11.79)	5.21***	(2.24,12.13)
Mother-to-child transmission knowledge	0.68	(0.36,1.27)	0.55	(0.23,1.29)
Pregnancy loss (ever)	0.40*	(0.23,0.71)	0.44*	(0.20,0.93)
Child loss (ever)	0.67	(0.40,1.10)	0.71	(0.27,1.87)
Perceived provider support for pregnancy and HIV (ref = little/no support)	1.21	(0.37,3.91)	1.25	(0.30,5.19)
Perceived partner would be upset about pregnancy (ref = supportive)	0.10*	(0.02,0.45)	0.13*	(0.03,0.63)
Don’t know	0.18*	(0.06,0.54)	0.33	(0.08,1.33)
Stigma scales				
HIV-internalized	1.60*	(1.26,5.70)	1.67	(0.86,3.24)
Sex work-internalized	1.30	(0.84,2.02)	1.47	(0.81,2.66)

* *p*-value< 0.05; ***p-value≤0.0001

For participants who reported not having a permanent contraceptive procedure, older age (OR:0.90; 95% CI: 0.86,0.95), having more children (OR:0.51; 95% CI: 0.36,0.72), detectable viral load (OR:2.30; 95% CI:1.07,4.92), negative perception of pregnancy and HIV (OR:5.21; 95% CI: 2.24,12.13), pregnancy loss (OR:0.44; 95% CI:0.20,0.93) and perception that pregnancy would upset their partner (OR:0.13; 95% CI:0.03,0.63) were associated with fertility desire. Living with HIV longer (OR:0.91; 95% CI:0.82,1.01) and ART use (OR:0.51; 95%CI:0.23,1.09) were marginally non-significant. HIV-internalized stigma (OR:1.67; 95% CI:0.86,3.24) was not significantly associated with fertility desire.

Table 4 highlights multivariate logistic regression results for both samples. Factors that retained significance for the total sample included age, number of children, positive perception of pregnancy and HIV, pregnancy loss, perceived partner feelings about pregnancy and HIV-related internalized stigma. Participants who were older (AOR: 0.94; 95% CI: 0.88,0.99) and who currently had more children (AOR 0.61; 95% CI: 0.44,0.84) had decreased odds of fertility desire. Those reporting a positive perception of pregnancy and HIV had increased odds of fertility desire (AOR: 6.49, 95% CI: 2.27,15.39), while participants reporting a pregnancy loss were less likely to want children than those who had not (AOR: 0.437; 95% CI: 0.17,0.84). Participants who felt their partners would be upset (AOR: 0.12; 95% CI: 0.02,0.66) or were unsure about a partner’s reaction (AOR: 0.14; 95% CI: 0.03,0.58) as compared to perceived partner support about pregnancy were less likely to desire children. Participants who reported a higher degree of HIV-related internalized stigma had increased odds for fertility desire (AOR: 3.19, 95% CI: 1.5,6.78).

**Table 4:** Multivariate logistic regression associations with fertility desire among female sex workers living with HIV

Sociodemographics	Total study sample (*n* = 239)		Participants not reporting permanent contraception (*n* = 118)	
	AOR	95% CI	AOR	95% CI
Age	0.94*	(0.88,0.99)	0.95	(0.88,1.03)
Education	0.96	(0.44,2.10)	1.10	(0.38,3.23)
Number of children	0.61*	(0.44,0.84)	0.61*	(0.38,0.98)
HIV and sex work				
Years HIV positive	0.94	(0.85,1.03)	0.96	(0.83,1.11)
Viral load (ref = undetectable) (*n* = 122)	1.08	(0.79,4.12)	1.90	(0.66,5.49)
Sexual and reproductive health				
Perception of pregnancy and HIV (ref = negative)	6.49***	(2.27,15.39)	3.72*	(1.23,11.16)
Pregnancy loss (ever)	0.37*	(0.17,0.84)	0.67	(0.22,1.77)
Perceived provider support for pregnancy and HIV (ref = little/no support)	1.26	(0.28,5.67)	1.09	(0.15,7.53)
Perceived partner would be upset about pregnancy (ref = supportive)	0.12*	(0.02,0.66)	0.13*	(0.02,0.81)
Don’t know	0.14*	(0.03,0.58)	0.25	(0.04,1.37)
Stigma scale				
HIV-internalized	3.19*	(1.5,6.78)	3.29*	(1.21,8.94)

* *p*-value< 0.05; ***p-value≤0.0001

For participants not reporting a permanent contraceptive procedure, number of current children (AOR:0.61; 95% CI:0.38,0.98), positive perception of pregnancy and HIV (AOR:3.72; 95% CI:1.23,11.16), perception that partner would be upset by a pregnancy (AOR:0.13; 95% CI:0.02,0.81) and greater HIV-internalized stigma (AOR:3.29; 95% CI: 1.21,8.94) all maintained significance in the final model. Older age (AOR:0.95; 95% CI:0.88,1.03), pregnancy loss (AOR:0.67; 95% CI:0.22,1.77) and being unsure of partner’s reaction to pregnancy (AOR:0.25; 95% CI:0.04,1.37) were not significantly associated with fertility desire.

## Discussion

Many gaps exist in the literature surrounding the reproductive health needs of marginalized populations of reproductive age [34]. As our findings show, pregnancy, childbearing and fertility desire are common among female sex workers living with HIV in this setting. Most participants had multiple children and pregnancies and many reported pregnancy and fertility desire after their HIV diagnosis, highlighting the importance of non-judgmental and integrated reproductive health services. The high prevalence of pregnancy loss and number of participants whom reported both fertility desire and permanent contraception raises questions about reproductive health rights, experiences and access to reproductive health services. The influence of stigma on reproductive health decision-making highlights the importance of multi-level interventions for sex workers living with HIV.

### Permanent contraception and fertility desire

About half of the population had undergone a permanent contraceptive procedure and 34.3% of participants who reported permanent contraception still desired children, which is of significant concern. Sterilization rates are historically high in the DR, with about 40.9% of women reporting sterilization in the general population [35]. The high levels of sterilization are associated with the length of time this practice has been offered in the DR, which became a normalized procedure over time [27]. Providers in the DR have reported emphasizing sterilization when counseling WLHA on family planning [24]. While there is limited information on sterilization regret, one study found that some women in the general population in the DR did not fully understand the permanence of sterilization and indicated regret [27]. Further, Human Rights Watch found the women living with HIV in the DR have been sterilized because of their HIV status without receiving full information about their choices and rights [26], as seen in other settings [36]. It is not known whether women who had undergone permanent contraception understood the consequence of their procedure or if they simply indicated preference for more children with the knowledge that they were not able to have children. However further research should explore this serious issue, especially patient-provider communication about contraceptive choices among WLHA as well as FSWs living with HIV/AIDS as there is a significant need for non-discriminatory and appropriate counseling on family planning and contraception with their providers.

Associations with fertility desire did not differ greatly between the sample population who reported not having a permanent contraceptive procedure as compared to the total study population. The association between HIV internalized stigma and fertility desire, however, was not significant in the bivariate analysis among those reporting no permanent contraception, although it was significant in the final model. Participants who reported a permanent contraceptive procedure may have been exposed to greater HIV-related stigma from health providers than those who did not, particularly if the procedure was done due to their HIV status- as reported in prior studies [36]. Further research is needed on experiences with stigma and discrimination when accessing reproductive care for this population.

### Stigma and fertility desire

Motherhood among women living with HIV and other marginalized populations has a positive impact on life aspirations, self worth, responsibility [3, 5, 6] and social acceptance [2, 7]. For a population dealing with multiple layers of stigma, having children may increase feelings of acceptance and pride, particularly in the DR where importance is placed on motherhood and family. Interestingly, sex work internalized stigma was not significantly associated with fertility desire. Many participants were part of sex worker support organizations, therefore may have felt less occupational stigma. More research is needed to understand specific associations between fertility desire and sex workers living with HIV, particularly in areas where sex work is criminalized. Qualitative studies and comparative studies that focus on sex work characteristics could provide a greater understanding of important influences. Additionally, many participants had a negative perception of pregnancy among women with HIV, further indicating internalization of HIV-related stigma and its impact on fertility desire. The influence of perceived partner’s reaction to pregnancy on fertility decisions has been found in other studies [5], highlighting the importance of engaging partners in reproductive health decision-making. These findings add to the importance of multi-level interventions focused on stigma and integrated care as central a central component for care and treatment for sex workers living with HIV as indicated in the literature more broadly [37].

### ART, viral load and fertility desire

In the bivariate analysis participants who reported current ART use were half as likely to want children and those with a detectable viral load were twice as likely to want children, although both variables lost significance in the final model. Interestingly, an undetectable viral load can influence increased physical health and indicate access/adherence to treatment, both of which has shown to increase fertility desire [5]. Associations between ART and fertility desire have varied in the literature by setting among women with HIV. A pooled meta-analysis between pregnancy desire and ART showed no significant associations [38], however a systematic review on fertility desires found positive associations with ART access [5]. These results may indicate exposure to negative attitudes from healthcare services about pregnancy and HIV, as those who were virally suppressed and on treatment are more likely accessing services and less likely to want children. A study on women living with HIV found that perceived or experienced stigma from health providers resulted in more negative attitudes towards childbearing [39]. However, further research is needed to understand specific associations with fertility desire.

### Fertility and childbearing

Fertility desire in this population is only slightly less than women in the general population in the DR, at 35.8% [40] and similar to sex workers in other studies- pregnancy intent was 27.5% in a Canadian study and intent to conceive was 19.8% in Burkina Faso and Togo [41]. Women living with HIV also range in fertility desire, from about 20 to 47% globally [5]. In a country with strict abortion laws, 64% of those ever pregnant reported at least one pregnancy loss. This may be due to barriers in accessing reproductive health services or challenges to patient-provider communication about reproductive health. Although pregnancy loss could be due to multiple factors and could be intended or unintended, this high level of pregnancy loss is noteworthy.

### Limitations

There are number of limitations to this study. This cross-sectional study highlights associations at one time point, as only baseline information was available at the time. Female sex workers were generally recruited through peer navigators, which may create selection bias and may not be representative of other FSW living with HIV in the DR. Many questions were self- report, which may introduce respondent bias including pregnancy loss, child loss and permanent contraception. Participants were also asked to report of the year of their permanent contraceptive procedure to increase the validity of the measure. This study focused on fertility desire, therefore intent cannot be assumed. Additionally, the study’s overall sample size was not calculated based on the analysis conducted for this paper potentially limiting our ability to detect significant associations.

## Conclusions

These findings emphasize the need for further research and interventions targeting stigma and partner dynamics among female sex workers living with HIV to improve their reproductive health. The high prevalence of pregnancy loss, sterilization, and pregnancies after HIV diagnosis and the association between HIV internalized stigma and fertility desire further adds to the importance of greater interventions and research on the reproductive health of this population. Health providers can play a crucial role in promoting non-stigmatizing conversations about fertility desires and assist in engaging partners as appropriate. At a time when women living with HIV can have a safe and healthy pregnancy, those who desire children, particularly those at greatest risk for negative health outcomes such as female sex workers living with HIV, should be given the attention they need to ensure their reproductive rights and well-being.

## Abbreviations

COIN: Centro de Orientacion e Investigacion Integral
DR: Dominican Republic
IDCP: Instituto Dermatalogico y Cirugia de Piel Dr. Humberto Bogart Diaz
MODEMU: Movimiento de Mujeres Unidas
PCR: Polymerase chain reaction
